# Examining Social Isolation as a Mediator for the Environmental Influence on Cognitive Functioning

**DOI:** 10.1093/geroni/igab046.1465

**Published:** 2021-12-17

**Authors:** Kexin Yu

**Affiliations:** USC, LA, California, United States

## Abstract

Environments serve as one of the most pervasive stimuli to the brain, shaping cognitive functionality through neuroplasticity. Both the physical and social environments of living could affect the cognitive functioning of older adults. Nonetheless, the mechanisms on how the environments “get under the skin” are unknown. This study examines the potential mediating effect of social isolation on the relationships between environment and cognitive health. Wave 9 data from the National Healthy Aging Trend Study was employed. The working sample includes 2,313 older adults. Path analysis results showed that in-home disorder was positively related to social isolation. A more cohesive social environment was negatively related to social isolation. Higher social isolation scores were associated with worsen global cognitive functioning. In-home disorder, community disorder, and social environment had significant direct effects on cognitive health after adjusting for the mediating effect. Social Isolation partially mediates the environmental influence on cognitive functioning.

